# Evidence of viral survival in representative volumes of feed and feed ingredients during long‐distance commercial transport across the continental United States

**DOI:** 10.1111/tbed.14057

**Published:** 2021-03-24

**Authors:** Scott Dee, Apoorva Shah, Cassandra Jones, Aaron Singrey, Dan Hanson, Roy Edler, Gordon Spronk, Megan Niederwerder, Eric Nelson

**Affiliations:** ^1^ Pipestone Applied Research Pipestone Veterinary Services Pipestone MN USA; ^2^ SAM Nutrition Eden Prairie MN USA; ^3^ Department of Animal Sciences and Industry Kansas State University Manhattan KS USA; ^4^ Department of Veterinary and Biomedical Sciences South Dakota State University Brookings SD USA; ^5^ Department of Diagnostic Medicine/Pathobiology College of Veterinary Medicine Kansas State University Manhattan KS USA

**Keywords:** animal feed, long distance, swine, transport, viral diseases

## Abstract

The hypothesis that feed ingredients could serve as vehicles for the transport and transmission of viral pathogens was first validated under laboratory conditions. To bridge the gap from the laboratory to the field, this current project tested whether three significant viruses of swine could survive in feed ingredients during long‐distance commercial transport across the continental US. One‐metric tonne totes of soybean meal (organic and conventional) and complete feed were spiked with a 10 ml mixture of PRRSV 174, PEDV and SVA and transported for 23 days in a commercial semi‐trailer truck, crossing 29 states, and 10,183 km. Samples were tested for the presence of viral RNA by PCR, and for viable virus in soy‐based samples by swine bioassay and in complete feed samples by natural feeding. Viable PRRSV, PEDV and SVA were detected in both soy products and viable PEDV and SVA in complete feed. These results provide the first evidence that viral pathogens of pigs can survive in representative volumes of feed and feed ingredients during long‐distance commercial transport across the continental United States.

## 1 INTRODUCTION

In 2014, it was first reported that pigs could become infected with porcine epidemic diarrhoea virus (PEDV) following consumption of contaminated feed via natural feeding behaviour (Dee et al., 2014). Since that time, similar observations have been reported for Seneca virus A (SVA), porcine reproductive and respiratory syndrome virus (PRRSV) and African swine fever virus (ASFV) (Dee et al., 2020a; Niederwerder et al., 2019,). These and other studies have also confirmed that certain feed ingredients, that is, soy‐based products, are protective to viruses and enhance their survival for extended periods under simulated conditions of transoceanic shipping. (Dee et al., 2015, 2016, 2018; Stoian et al., 2020).

In support of these laboratory‐based findings, a demonstration project was conducted to evaluate survival of viruses in feed ingredients under real‐world shipping conditions (Dee et al.,  2020b). Thirty‐gram samples of several feed ingredients feed were spiked with 2‐mL mixture of PRRSV 174, PEDV and SVA and transported for 21 days in the trailer of a commercial transport vehicle, crossing 14 states and over 9,741 km. While the study successfully demonstrated that infectious PRRSV, PEDV and SVA were present in both soy products, it possessed inherent limitations as the experimental design did not accurately represent the commercial trucking industry or the commercial feed industry, since it utilized very small volumes of feed (30g) which did not accurately portray the challenges associated with testing bulk ingredients. In addition, the 30‐g samples were inoculated with relatively large volumes of liquid (2ml per sample), and viral viability was only assessed via swine bioassay and an evaluation of viral transmission via natural feeding behaviour was not included.

To address these acknowledged limitations, we conducted a new study to better bridge the gap between the laboratory and the field. The experimental design incorporated several characteristics of the commercial trucking and feed industries, that is, the use of semi‐trailer truck and a commercial route of transit, along with the use of larger volumes of feed, which were sampled using a standardized method for the testing of bulk feed. In addition, a viral challenge designed to simulate a ‘hot spot’ of contamination, as seen with aflatoxin contamination of grain, was used, and both viability and transmission were assessed via bioassay and natural feeding behaviour. The study was based on the hypothesis that certain viruses can survive in select feed and feed ingredients during long‐distance commercial transport under real‐world conditions.

## 2 MATERIALS AND METHODS

### 2.1 Animal care and use

Pigs used in the study were housed in the Pipestone Applied Research biosafety level 2 facility in accordance with the institutional animal care and use guidelines approved by the investigators ethical review board (Pipestone Applied Research IACUC trial number 2021–01).

### 2.2 Feed preparation

Types of feed used in the study included conventional soybean meal, (1 to 2% fat and 46 to 47% protein), organic soybean meal, (6 to 7% fat and 44 to 45% protein) (Dee et al., 2016, 2018) and complete grow‐finish swine feed. These ingredients were added in bulk to new polypropylene bags, each with a capacity of 1.74 m^3^ (National Bulk Bag, Champlin, MN, US), resulting in totes with a final volume of 1‐metric tonne per tote. For this study, two 1‐metric tonne totes of conventional soybean meal, two 1‐metric tonne totes of organic soybean meal and three metric tonnes of complete feed, seven totes in total, were prepared. Totes were then delivered to a dispatching point in Fridley, MN, to prepare for embarkation.

### 2.3 Tote inoculation

To simulate a ‘hot spot’ model of feed contamination, 10‐mL ice cubes containing a mixture of PRRSV 174, PEDV and SVA at a total dose of 1 x 10^5^ TCID_50_ per virus was prepared. Each virus was diluted in 30 ml of minimum essential medium (MEM, Sigma‐Aldrich, St. Louis, MO, USA) to a concentration of 1 x 10^5^ TCID_50_/mL per virus and mixed (three viruses for a total of 90 ml) followed by an addition of 210‐mL MEM, to bring the total volume to 300 ml. Ice cubes were prepared by freezing 10‐mL aliquots of the mixture in 50‐mL conical centrifuge tubes (Corning Inc. Corning, NY, USA) at −80^0^C. Six totes, two containing conventional soybean meal, two containing organic soybean meal and two containing complete feed, were inoculated. The final tote of complete feed was used as a negative control.

To inoculate the six designated totes, a previously filled tote was elevated using a forklift and placed 15 cm directly above an empty tote, with its duffle top held open in a fixed position. The spout bottom of the upper tote was then opened, allowing feed to flow via gravity into the opening of the empty tote. When the lower tote was approximately half full, an ice cube containing the described viral mixture was blindly dropped into the lower tote. The remainder from the upper tote was then added to the lower tote, burying the cube from sight, and the duffle top was tied shut after the lower tote was filled to completion.

### 2.4 Controls

For controls, twelve 30‐g allotments of feed (four conventional soybean meal samples, four conventional organic soybean meal samples and four complete feed samples) were weighed into individual 50‐mL mini‐bioreactor tubes with vented caps. Six of the twelve samples were individually spiked with a 2‐mL aliquot from the viral mixture described previously, to serve as positive controls. The aliquot was injected directly into the centre of each 30‐g ingredient sample using a 3‐mL syringe with an 18‐gauge, 3.81‐cm needle. The remaining six samples (30g‐feed, no virus) served as negative controls.

### 2.5 Details of transport

#### 2.5.1 Transport vehicle

To transport the seven totes and control tubes, a commercial semi‐trailer truck with a 15.8‐m trailer was used (Csp Delivery, Fridley, MN, US). Totes on pallets were moved into the proximal end of the trailer using a forklift. Control samples were stored in a box on the trailer floor, surrounded by the totes. To record temperature and relative humidity (% RH) level in totes during the trip, a data logger (RC‐51H, ELITech, Paris, FR) was placed inside one of the conventional soybean meal totes and one of the organic soybean meal totes at the 50% point of filling in the centre of the tote. These instruments recorded temperature and % RH every 15 min during transit. In addition, a GPS system within the transport vehicle was used to track location, time in transit and distance travelled.

### 2.6 Details of travel plan

**FIGURE 1:**
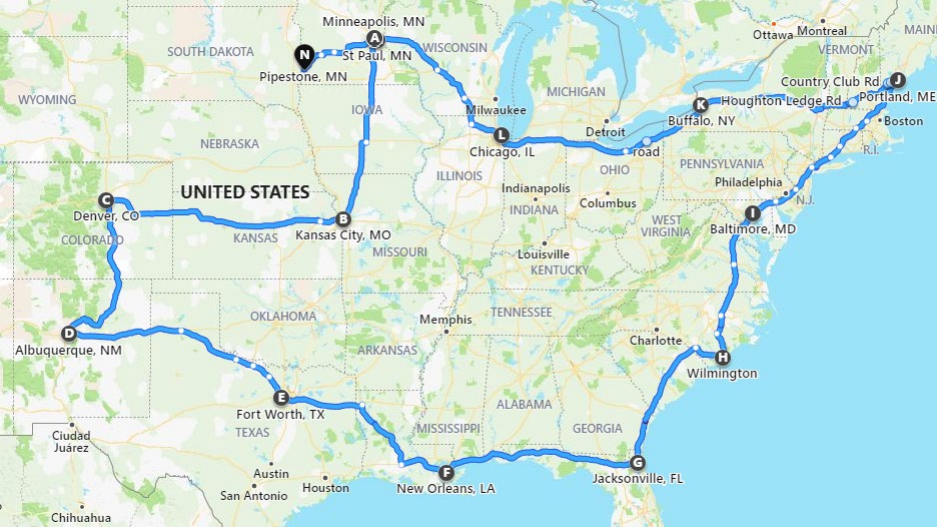
Map displaying the route travelled during the study

The study utilized a route of delivery representative of the commercial trucking industry which involved travel through 29 US states. The goal of this route was to cover several regions of the United States and expose the feed ingredients and viruses to a wide variety of environmental conditions. The route was initiated in Minneapolis, Minnesota, and travelled through Iowa to Kansas City, Missouri (overnight stay), across Kansas to Denver, Colorado (overnight stay), to Albuquerque, New Mexico (overnight stay), to Fort Worth, Texas (overnight stay), and then to New Orleans, Louisiana (overnight stay). Travel continued along the Gulf Coast across the states of Mississippi, Alabama and Georgia into Jacksonville, Florida (overnight stay), and proceeded up the eastern seaboard through South Carolina to Wilmington, North Carolina (overnight stay), through Virginia, up to Baltimore, Maryland (overnight stay), through Delaware, New Jersey, passing through New York City on the way to Connecticut, Massachusetts and New Hampshire and up to Portland, Maine (overnight stay). The truck then returned to the Midwest through New Hampshire and Vermont, to Buffalo, New York (overnight stay), travelling through the states of Pennsylvania, Ohio, Indiana to Chicago, Illinois (overnight stay), then through Wisconsin to Minneapolis, Minnesota, finally stopping in Pipestone, Minnesota. Figure 1 provides a map summarizing the route with the overnight cities highlighted.

### 2.7 Quality control and project oversight

Prior to the study, details of the project were relayed to the Food and Drug Administration Center for Veterinary Medicine (FDA CVM), the United States Department of Agriculture, and to the directors of the respective boards of animal health in states where the truck and trailer were planning to stay overnight. Totes were labelled per FDA CVM instructions stating that feed was ‘Not for human or animal consumption/for research use only’, and a letter of approval from the agency, co‐signed by the PI with respective contact information was carried by the driver during the entire trip. Prior to departure, a barrier was inserted into the trailer to secure the totes within the proximal half of the trailer to minimize the risk of tote movement, spillage, etc., during transport. Other than the seven totes and the controls, no other products were included on the trailer. The truck did not plan to stop anywhere during the transit period, other than for refuelling, meals and hotel stays.

### 2.8 Bulk sampling and processing

**FIGURE 2:**
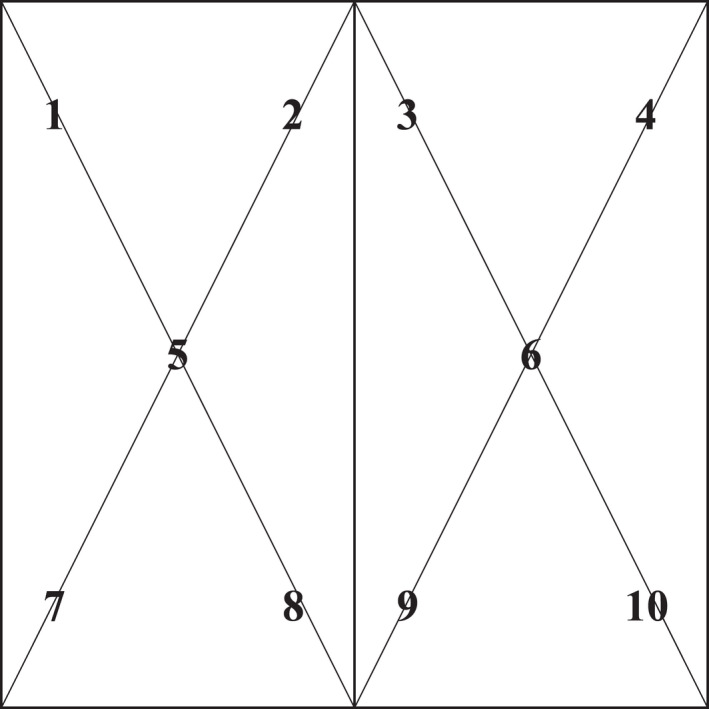
Schematic of AAFCO protocol for sampling bulk ingredients used in the study. Ten samples were collected from each tote using two ‘X’ patterns resulting in a composite sample per tote

On day 0 and day 23 post‐inoculation, the seven totes were sampled using a method based on the Association of American Feed Control Officials (AAFCO) Feed Inspector's Manual, which had recently been validated for detection of PEDV in feed (Jones, Stewart, Woodworth, Dritz, & Paulk, 2020a). Totes were sampled using a 0.99‐cm long stainless‐steel grain probe with an open handle and six openings (Seedburo Equipment Co., Des Plaines, IL, US). According to protocol, 10 samples were collected from each tote using two ‘X’ patterns (AAFCO, 2014) and then mixed in a 1‐litre plastic bag (Ziploc, S.C. Johnson & Son, Racine, WI, US) to create a single composite sample (Figure 2). After each tote was sampled, feed dust was expelled from the probe using forced air, sprayed with 70% ethanol, wiped with a clean cloth and the ethanol allowed to evaporate prior to the next sampling. Gloves were also changed between every tote. Following collection, samples from the four soy‐based products were processed to prepare inoculums for PCR and bioassay testing. Specifically, each soy‐based bulk feed sample collected from its respective tote was mixed with 1,000 ml of sterile phosphate‐buffered saline in a 4‐litre metal can, the can sealed, inverted, shaken vigorously by hand and then placed on a pneumatic paint shaker (Astro pneumatic tool 4,550, Astro pneumatic tool company, South el Monte, CA, US). Each mixture was shaken for two minutes, the liquid decanted into 250‐mL sterile plastic tubes, centrifuged at 4000*g* for 10 min, supernatant decanted into 50‐mL sterile plastic tubes and recentrifuged at 4000*g* for 10 min. The four samples were frozen at −80^0^ C in preparation for testing and inoculation. For testing of the positive control samples, each of the six samples was added into a 250‐mL conical tube, followed by the addition of 60 ml of sterile saline. The sample was then homogenized and centrifuged 4000*g* for 10 min, with supernatant decanted into a clean 50‐mL tube and recentrifuged at 4000*g* for 10 min. Supernatant was then decanted into 10‐mL tubes and frozen at −80^0^ C, in preparation for testing and inoculation.

### 2.9 Diagnostic testing

Following processing, samples were evaluated for the presence of viral RNA by PCR and for virus viability by swine bioassay for soy‐based products and by natural feeding behaviour for complete feed. For PCR, samples were tested at the South Dakota State University Animal Disease Research and Diagnostic Laboratory (SDSU ADRDL) using published methods (Dee et al., 2014, 2015, 2016). For viability testing, pigs were housed in the Pipestone Research biosafety level 2 facility for a 21‐day period. For testing of soy‐based ingredients by swine bioassay, 24 five‐week‐old pigs, originating from a farm known to be naïve for PRRSV, PEDV and SVA were housed in a single room, four pigs per pen, across six pens. As the experimental unit for the study was the pen, pens were organized according to ingredient, specifically: pen 1 represented conventional soybean meal tote one, pen 2 represented conventional soybean meal tote two, pen 3 represented organic soybean meal tote one, pen 4 represented organic soybean meal tote two, pen 5 represented positive control samples, while pen 6 represented negative control samples. Solid pen dividers were placed between pens to prevent nose‐to‐nose contact between groups and minimize cross‐contamination. For the assessment of viable virus in conventional soybean meal tote one, the four pigs in pen one were each inoculated with 2 ml via the intramuscular route, 2 ml via the oral route and 2 ml via the intranasal route. For the assessment of viable virus in conventional soybean meal tote two, four pigs in pen two were each inoculated with 2 ml via the intramuscular route, 2 ml via the oral route and 2 ml via the intranasal route. The same inoculation procedures were followed for samples of from organic soybean meal totes (pen 3 and pen 4). All six positive control samples (two samples from conventional soy totes, two samples from organic soy totes and two samples from complete feed totes) were pooled, and the four pigs in pen 5 were inoculated as described. Finally, all six negative control samples were pooled, and pigs in pen 6 were inoculated as described. For the testing of complete feed for the presence of viable virus, the three totes of complete feed from the transport vehicle were loaded into a single feed bin and 18–100 kg pigs, originating from the same source as the bioassay pigs, were housed in a single room (six pens, three pigs per pen) and allowed to consume the complete feed via natural feeding behaviour. During the 21‐day period, a pen‐based sampling protocol was employed to determine the status of each pen using oral fluid samples that were collected from each of the 12 pens on days 0, 7 and 14 post‐inoculation. To support results from the oral fluid samples, clinically affected pigs were humanely euthanized, and tonsil tissue, rectal swabs and blood samples collected. All samples were tested by PCR at the SDSU ADRDL.

### 2.10 Data analysis

Temperature and % RH data from the two soybean meal totes collected during the transport period were summarized using descriptive statistics. Differences in mean temperature and mean % RH between the conventional soybean meal and the organic soybean meal were analysed for significance using a two‐sample *t*‐test.

## 3 RESULTS

### 3.1 Summary of the transport period

The transport period took place over 23 days, from November 30, 2020, to December 22, 2020. The route covered 29 states, for a total of 100.2 hr in transit over 10,183 km (Figure 1). The truck and its cargo travelled through the Midwest region, the Rocky Mountain region, the Southwest region, the Gulf Coast, the Eastern Seaboard, the New England region and the Great Lakes region. No accidents, unexpected stops or changes to the itinerary occurred throughout the journey.

### 3.2 Feed samples

The mean temperature of the conventional soybean meal and the mean temperature of the organic soybean meal were significantly different (*p* <.0001) from one another, as were the mean % RH of the conventional soybean meal and the mean % RH of the organic soybean meal (*p* <.0001) (Table 1). A total of 14 composite samples were collected across the seven totes, seven samples on day 0 and seven samples of day 23. The mean weight per composite sample was 1.04 kg, with a range of 0.91 kg to 1.4 kg.

**TABLE 1:** Temperature (T) and % relative humidity (RH) data collected from probes placed inside two of the totes during the transport period

Location of probe	# datapoints	Mean T	Max T	Min T	Mean RH	Max RH	Min RH
SBM‐C (inside filled tote)	2,132	9.4^0^C**^a^**	17.0^0^C	3.2^0^C	66%**^a^**	68%	38%
SBM‐O (inside filled tote)	2,132	7.9^0^C**^b^**	17.5^0^C	1.0^0^C	21%**^b^**	37%	20%

Note

Difference in superscripts (a/b) indicates a difference in significance of *p* <.05.

SBM‐C/SBM‐O: conventional or organic soybean meal.

Inside filled tote: probe was inserted inside of the tote at the point when the tote was 50% filled.

### 3.3 Presence of viral nucleic acid in feed

The results of the PCR testing of samples from the totes are summarized in Table 2a. Across the six inoculated totes and the three viruses in the inoculum, viral RNA was detected in 67% (12/18) of the day 0 samples. Of the 12 positive samples, 50% (3/6) were positive for PEDV RNA, 100% (6/6) were positive for SVA RNA and 50% (3/6) were positive for PRRSV RNA. On day 23 post‐inoculation, viral RNA was detected in 50% (9/18) of inoculated tote samples with 50% (3/6) of the samples positive for PEDV RNA, 67% (4/6) of the samples positive for SVA RNA and 33% (2/6) of the positive for PRRSV RNA. All samples from the negative control complete feed tote were PCR negative at both sampling points. The per cent detection in soy‐based products across all viruses was 83% (10/12 samples positive) on day 0 and 75% (8/12 samples positive) on day 23, with all samples from soybean meal organic tote one PCR negative. In contrast, RNA detection in complete feed was 33% (2/6 samples positive) on day 0 and 17% (1/6 of the samples positive) on day 23, with SVA the only virus detected. Regarding the positive controls, conventional soybean meal samples and organic soybean meal samples were PCR positive across all three viruses on day 23. Control samples of complete feed were PCR positive for PEDV RNA and SVA RNA and negative for PRRSV RNA on day 23. Finally, all negative control samples were PCR negative (Table 2b).

**TABLE 2a:** PCR results from bulk ingredient sampling on day 0 and day 23 post‐inoculation

Ingredient^a^	DPI^f^	PEDV Ct	SVA Ct	PRRSV Ct	DPI	PEDV Ct	SVA Ct	PRRSV Ct
SBM‐C−1^b^	0	**37.8**	**34.8**	**33.5**	23	**34.8**	**35.6**	neg
SBM‐C−2	0	**37.2**	**33.2**	**32.6**	23	**34.4**	**35.7**	**34.9**
SBM‐O−1^c^	0	**35.1**	**34.6**	**34.5**	23	neg	neg	neg
SBM‐O−2	0	neg	**36.2**	neg	23	**37.6**	**35.3**	**34.1**
CF−1^d^	0	neg	**36.1**	neg	23	neg	neg	neg
CF−2	0	neg	**35.1**	neg	23	neg	**35.5**	neg
CF (‐) control^e^	0	neg	neg	neg	23	neg	neg	neg

^a^ one‐metric tonne tote batches.

^b^ conventional soybean meal tote 1 or 2 (inoculated).

^c^ organic soybean meal tote 1 or 2 (inoculated).

^d^ complete feed tote 1 or 2 (inoculated).

^e^ complete feed (uninoculated).

^f^ days post‐inoculation.

**TABLE 2b:** PCR results of positive and negative control pools on day 23 post‐inoculation

Ingredient	DPI	PEDV Ct	SVA Ct	PRRSV Ct
SBM‐C−1‐pos^a^	23	**27.8**	**27.1**	**25.5**
SBM‐C−2‐pos	23	**27.2**	**26.2**	**24.8**
SBM‐C neg	23	neg	neg	neg
SBM‐O−1‐pos^b^	23	**27.1**	**27.8**	**26.2**
SBM‐O−2‐pos	23	**27.2**	**27.7**	**25.8**
SBM‐O‐neg	23	neg	neg	neg
CF−1‐pos^c^	23	**35.1**	**27.4**	**34.0**
CF−2‐pos	23	**36.2**	**27.7**	**35.7**
CF neg	23	neg	neg	neg

^a^ SBM‐C‐1/SBM‐C‐2: conventional soybean meal tote 1/ conventional soybean meal tote 2.

^b^ SBM‐O‐1/SBM‐O‐2: organic soybean meal batch 1/: organic soybean meal batch 2.

^c^ CF‐1/CF‐2: complete feed tote 1/complete feed tote 2.

### 3.4 Presence of viable virus in feed

Prior to inoculation, all pigs were confirmed to be naïve to all three viruses via oral fluid samples collected on day 0. Following inoculation, PRRSV, SVA and PEDV infection was confirmed by the presence of PCR‐positive oral fluid samples detected across both conventional soybean meal pens and one organic soybean meal pen (Table 2c). Similar results were obtained from the positive control pen, while samples from the negative control pen were negative. Clinical signs suggestive of PRRSV (dyspnoea and hyperthermia), PEDV (diarrhoea) and SVA (lameness) were observed in pens across both soy groups and the positive controls. In addition, serum (PRRSV), tonsil tissue (SVA) and rectal swabs (PEDV) were PCR positive in one clinically affected pig from the conventional soybean meal pen, the organic soybean meal pen and the positive control pen. Regarding pigs in the natural feeding behaviour group, SVA and PEDV infection were confirmed by the presence of PCR‐positive oral fluid samples in two of six pens. Clinical evidence of lameness, diarrhoea and weight loss was observed in animals in these pens, and PEDV RNA and SVA RNA were detected in tissue samples from one pig in both pens. All samples were negative for PRRSV RNA (Table 2c).

**TABLE 2c:** Pen‐based oral fluid results by ingredient and virus following inoculation with samples collected on day 23 of the transport period

Ingredient	Viability Assay	Pen	PEDV	SVA	PRRSV	Necropsy Confirmation
SBM‐C−1^a^	bioassay	1	POS^e^	POS	NEG	YES^g^
SBM‐C−2^a^	bioassay	2	POS	NEG	POS	YES
SBM‐O−1^b^	bioassay	3	NEG^f^	NEG	NEG	NA^h^
SBM‐O−2^b^	bioassay	4	POS	POS	POS	YES
(+) controls^c^	bioassay	5	POS	POS	POS	NA
(‐) controls^c^	bioassay	6	NEG	NEG	NEG	NA
Complete feed^d^	natural feeding	7	POS	POS	NEG	YES
Complete feed	natural feeding	8	POS	NEG	NEG	YES
Complete feed	natural feeding	9	NEG	NEG	NEG	NA
Complete feed	natural feeding	10	NEG	NEG	NEG	NA
Complete feed	natural feeding	11	NEG	NEG	NEG	NA
Complete feed	natural feeding	12	NEG	NEG	NEG	NA

^a^ conventional soybean meal (SBM‐C) tote 1 or 2

^b^ organic soybean meal (SBM‐O) tote 1 or 2.

^c^ all six positive controls were pooled and all six negative controls were pooled.

^d^ totes of complete feed were pooled into one feed bin to facilitate natural feeding behaviour.

^e^ POS = positive detection of viral RNA in a pen‐based oral fluid sample.

^f^ NEG = lack of detection of viral RNA in a pen‐based oral fluid sample.

^g^ YES = necropsy results confirmed oral fluid results.

^h^ NA = necropsy confirmation not attempted.

## 4 DISCUSSION

The ability of feed and feed ingredients to serve as vehicles for the transport and transmission of viral pathogens is a relatively new area of science. Since the initial description of PEDV transmission in feed, an extensive amount of experimental evidence has been compiled to the point where comprehensive literature reviews can now be written on the topic and risk assessments can be conducted (Dee et al., 2020a, Jones et al., 2020b). Yet, to continue to challenge the hypothesis regarding the risk of feed, studies must be performed outside of the laboratory, utilizing experimental designs and conditions that recreate what happens every day in the field, under real‐world conditions. This project was the first attempt to evaluate virus survival over long distances under conditions experienced during a commercial transport event across the continental United States. It utilized an experimental design that incorporated real‐world elements, such as the use of a commercial transport vehicle, a route of transit that crossed multiple regions of the United States, a standardized method of bulk feed sampling, and an evaluation of viral genome, viability and transmission. The design was further strengthened by a novel challenge model designed to simulate a ‘hot spot’ of contamination, the use of proper controls, environmental monitoring, along with oversight and guidance from federal agencies.

This holistic approach advanced knowledge in three specific areas: 1) information on the temperature and % RH in feed totes during transit, 2) a better understanding of the strengths and limitations of the bulk sampling process, and 3) further data supporting the ability of certain viruses to survive in feed during real‐world transport events. Regarding point number 1, we now have preliminary insight into the levels of temperature and % RH present in feed totes during transit across the continental United States. Despite the small sample size, it was interesting to note the significant difference in both the mean temperature and the mean % RH in the organic soybean meal tote versus the conventional soybean meal tote. While this outcome is based on only two totes, a large number (2,132) of datapoints were collected from each tote, resulting in information that could be used to generate new hypotheses on why certain ingredients are protective to certain viruses. In the case of point number 2, it is the opinion of the authors that the bulk sampling protocol worked well overall, particularly, since we were blinded to the location of the ‘hot spot’. It was interesting to note that once again, soy‐based ingredients were protective to all three viruses, stabilizing both viral genome and viable virus. In contrast, complete feed was not as forgiving. For example, all complete feed samples were PEDV PCR negative at day 23 post‐inoculation; however, infection still occurred following natural feeding behaviour, suggesting that virus went undetected during sampling. Regarding SVA, RNA was detected in complete feed totes and infection was documented post‐feeding, once again demonstrating the ability of this virus to survive for extended periods in feed. In contrast, PRRSV RNA was not detected in samples from complete feed totes and infection did not take place following feeding, suggesting that PRRSV did not survive in this feed matrix during long‐distance transport, similar to what had been reported under laboratory conditions (Dee et al., 2018). Finally, as it pertains to point number 3, this study demonstrated the ability of all three viruses to survive and to be infectious to pigs following a 23 day period in commercial transit across the continental United States. Based on this outcome, we now have solid evidence that feed and feed ingredients can serve as vehicles for the transport and transmission of three significant viral pathogens of veterinary significance under real‐world conditions.

Despite these advancements, as with all experiments, this study had its share of acknowledged strengths and limitations. Strengths included the real‐world approach of the experimental design, including a representative route of transit involving tonnes of feed, exposure of viruses to the differing climates found in multiple regions across the United States, a blinded ‘hot spot’ challenge model that used a minimal amount of liquid, sampling of bulk feed using a grain probe‐based methodology and the use of natural feeding behaviour to assess transmission. Another strength was that biosecurity was maximized during transit, since no other products were included in the trailer, the seven totes were barricaded in the trailer to minimize movement during transport, and no stops were made at agricultural sites. In addition, this project involved a high level of state and federal input and oversight. Limitations centred primarily on sample size constraints, that is, only one replicate was conducted and only seven totes were sampled, with only two samples collected per tote. Therefore, the results from the study cannot be used to predict the frequency of any of the reported outcomes. While we acknowledge this limitation, to increase sample size would have required a fleet of trucks and numerous totes, issues that would have been both economically and logistically challenging. Finally, only a single viral concentration was used to inoculate totes and results may have been different at higher or lower doses of challenge.

In closing, we now have for the first time evidence of viral survival in representative volumes of feed and feed ingredients during an actual long‐distance commercial transport event across the continental United States. It is hoped that the information from this study, in combination with the current body of experimental evidence, will help to unify opinions across the swine industry, the veterinary profession and governmental agencies regarding the significance of the risk of feed for viral movement. Until we are united, we cannot make progress, and until that time, we all are at risk.

## CONFLICT OF INTEREST

The authors declare no conflict of interest.

## ETHICAL APPROVAL

Animals in this study were managed in accordance with the institutional animal care and use guidelines observed by the investigators’ ethical review board, Pipestone Applied Research IACUC, trial number 2021–01.

## Data Availability

All data from this study have been disclosed.
